# Supplementary figures and images for: Screening of Neutralizing Antibodies against FaeG Protein of Enterotoxigenic Escherichia coli
Source: Vet Sci. 2024 Sep 9;11(9):419. doi: 10.3390/vetsci11090419 (PMC11436151; doi:10.3390/vetsci11090419)

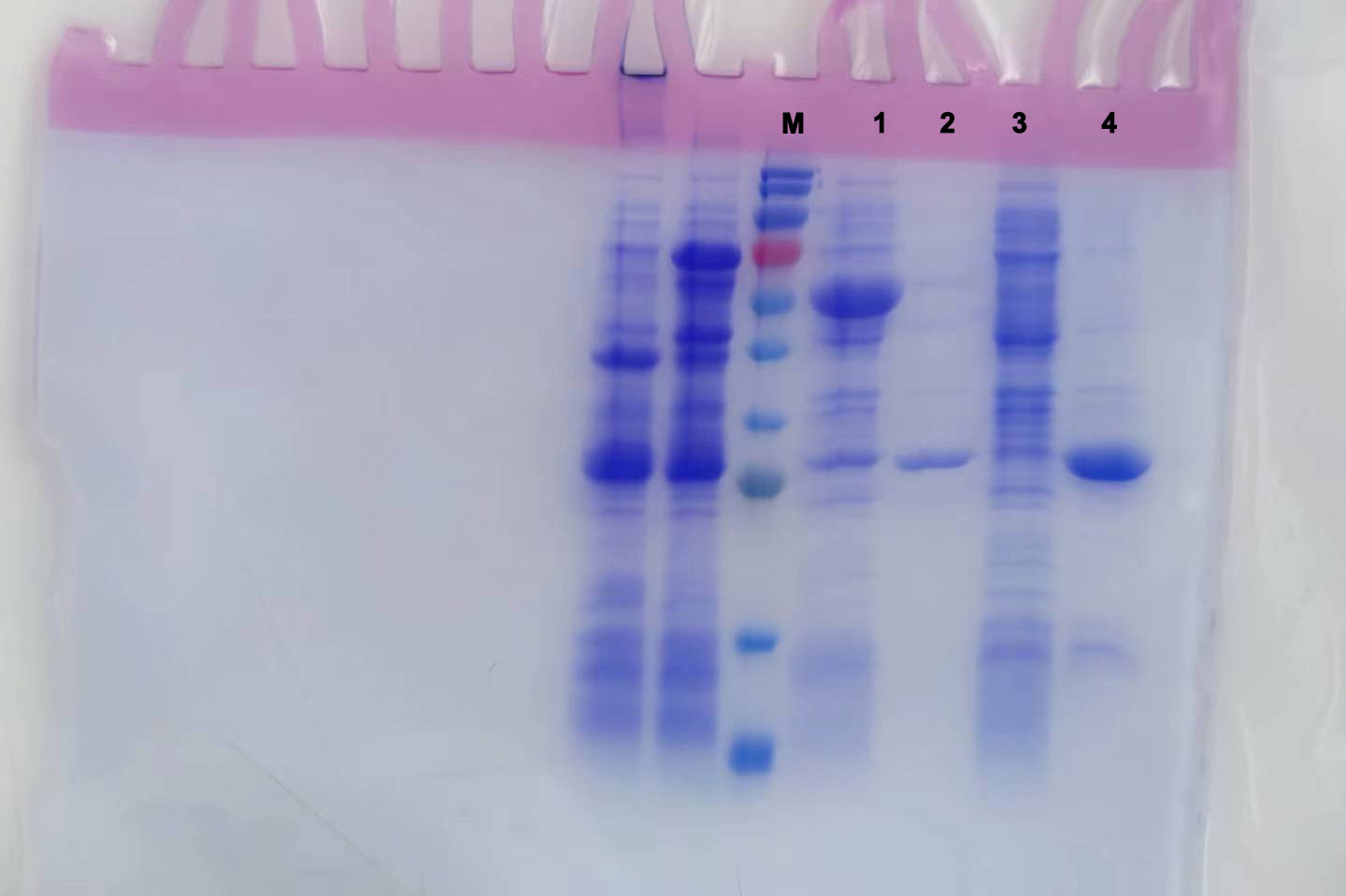

Supplement: Supplementary file 1 [file vetsci-11-00419-s001.zip › Figure S1A.jpg]

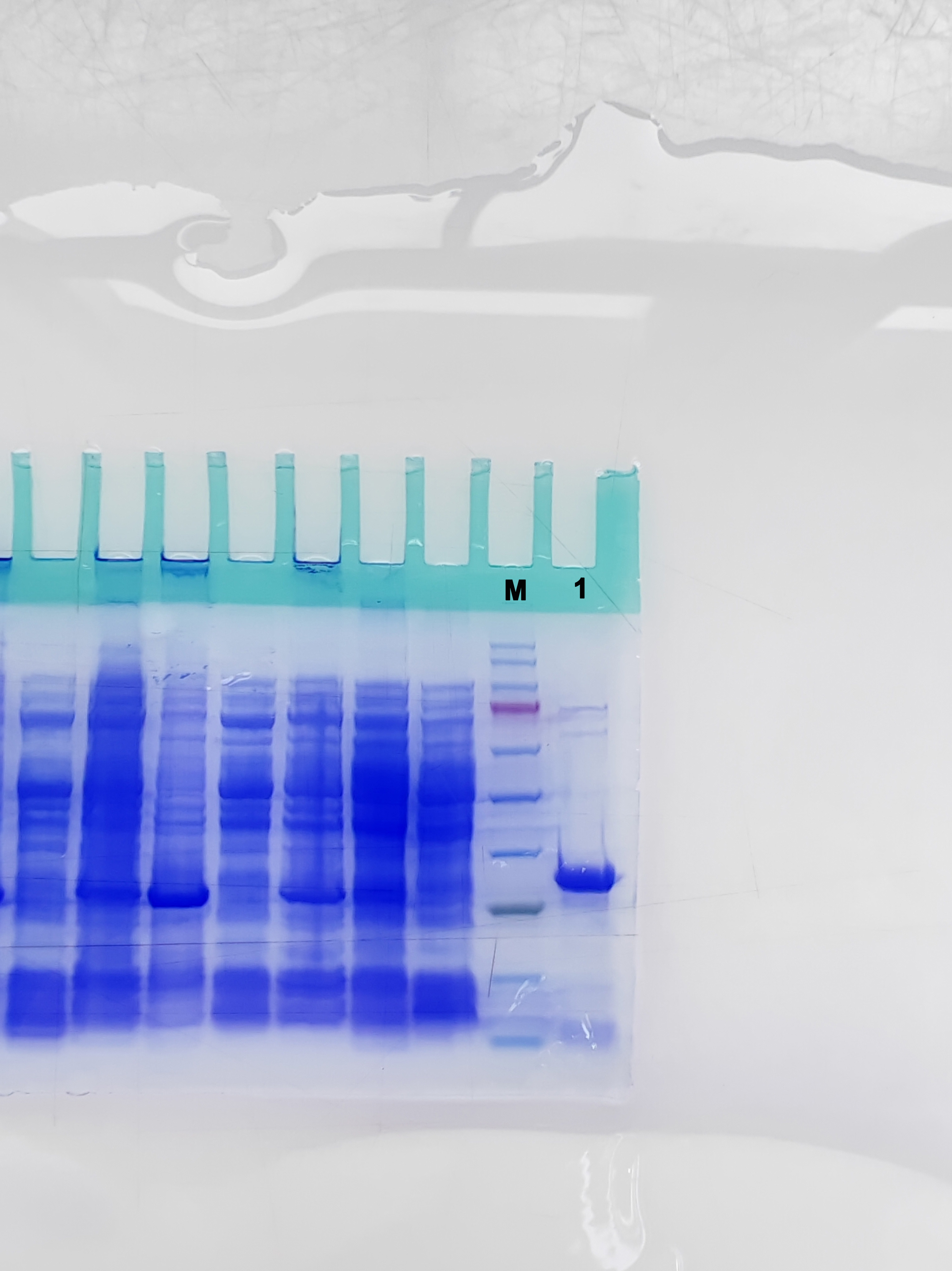

Supplement: Supplementary file 1 [file vetsci-11-00419-s001.zip › Figure S1B.jpg]

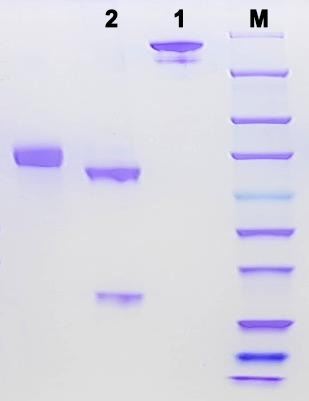

Supplement: Supplementary file 1 [file vetsci-11-00419-s001.zip › Figure S2B.jpg]
